# Improving antibiotic prescribing for adults with community acquired pneumonia: Does a computerised decision support system achieve more than academic detailing alone? – a time series analysis

**DOI:** 10.1186/1472-6947-8-35

**Published:** 2008-07-31

**Authors:** Kirsty L Buising, Karin A Thursky, James F Black, Lachlan MacGregor, Alan C Street, Marcus P Kennedy, Graham V Brown

**Affiliations:** 1 Victorian Infectious Diseases Service, The Royal Melbourne Hospital, Parkville, Victoria 3050, Australia; 2 Centre for Clinical Research Excellence in Infectious Diseases, Department of Medicine, University of Melbourne, Parkville, Victoria 3050, Australia; 3 Emergency Department, The Royal Melbourne Hospital, Parkville, Victoria 3050, Australia; 4 The Nossal Institute for Global Health, The University of Melbourne, Victoria, 3010, Australia

## Abstract

**Background:**

The ideal method to encourage uptake of clinical guidelines in hospitals is not known. Several strategies have been suggested. This study evaluates the impact of academic detailing and a computerised decision support system (CDSS) on clinicians' prescribing behaviour for patients with community acquired pneumonia (CAP).

**Methods:**

The management of all patients presenting to the emergency department over three successive time periods was evaluated; the baseline, academic detailing and CDSS periods. The rate of empiric antibiotic prescribing that was concordant with recommendations was studied over time comparing pre and post periods and using an interrupted time series analysis.

**Results:**

The odds ratio for concordant therapy in the academic detailing period, after adjustment for age, illness severity and suspicion of aspiration, compared with the baseline period was OR = 2.79 [1.88, 4.14], p < 0.01, and for the computerised decision support period compared to the academic detailing period was OR = 1.99 [1.07, 3.69], p = 0.02. During the first months of the computerised decision support period an improvement in the appropriateness of antibiotic prescribing was demonstrated, which was greater than that expected to have occurred with time and academic detailing alone, based on predictions from a binary logistic model.

**Conclusion:**

Deployment of a computerised decision support system was associated with an early improvement in antibiotic prescribing practices which was greater than the changes seen with academic detailing. The sustainability of this intervention requires further evaluation.

## Background

With the rapidly expanding body of medical knowledge, clinicians need access to appropriate, relevant information to guide their clinical decision making. For many conditions, clinical experts have used available evidence and experience to generate guidelines that endeavour to assist clinicians, and improve patient outcomes. A major problem, however, has been finding the best strategies to implement these guidelines in a busy hospital environment. [1-3] Group lectures, one to one academic detailing, laminated cards and advertising material such as posters have all been tried with variable success. [4-7] With the increasing role played by computers as a source of information in the hospital setting, computerised decision support may provide a useful alternate strategy. [8-11]

At the Royal Melbourne Hospital, a transferable web based computerised decision support system was developed, with the capacity to present any guideline or algorithm. [12] We chose in the first instance to deploy a guideline for the management of patients with community acquired pneumonia (CAP) as this is one of the most common conditions presenting to hospital emergency departments. International and national guidelines have been produced to guide the management of CAP [13-15], but uptake has been poor. [16]

The general aim of this study was to describe the impact of different methods of guideline promotion on clinician prescribing behaviour. More specifically, a comparison of the impact of both academic detailing (AD) and a computerised decision support system (CDSS) on the management of patients with CAP in an emergency department (ED) was examined. The outcomes of interest included the prescription of antibiotics that were concordant with guideline recommendations, the early identification of the severely ill patients and adjustment of antibiotics to meet recommendations for prescribing in the severely ill group, and adjustment of antibiotics to accommodate known patient allergies.

## Methods

### Design

A two stage pre and post intervention cohort study, and a time series analysis

### Setting

This study was performed at the Royal Melbourne Hospital, an urban adult tertiary teaching hospital with 350 beds including 14 intensive care unit (ICU) beds. The emergency department assesses 50,000 patients per year, leading to 16,000 admissions to hospital. This hospital did not have an electronic medical record or a computerised order entry system. Over 30 different doctors were working in the ED at any point in time over the study periods, and the allocation of doctors to patients was not structured. A computerised antibiotic approval system restricting access to ceftriaxone was also in operation over all three time periods of this study. Its implementation pre dated the commencement of this study. It approved ceftriaxone use for all patients with severe pneumonia, and its content agreed with the CAP guideline content.

### Participants

This study described the prescribing behaviour of doctors (both senior and junior medical staff) managing patients in the ED. Specifically, the study focused on antibiotic prescribing for all patients who were initially diagnosed with CAP by the treating clinician in the ED.

### Intervention

The study extended over three distinct time periods. The first, (or 'baseline') period was from April 2003–March 2004. The second (or 'academic detailing') period (AD) was February 2005–October 2005 and the third (or 'computerised decision support') period (CDSS) was from April 2006–September 2006.

During the first ('baseline') time period, electronic and paper copies of national antibiotic prescribing guidelines were available to staff in the ED [13] but no particular additional efforts were made to encourage uptake of the guideline.

At the start of the second ('academic detailing') time period, a program of academic detailing was initiated at the hospital. This involved training two senior ED clinicians, a pharmacist and a nurse to provide academic detailing to their colleagues. They spent one on one time educating colleagues (doctors and pharmacists) about antibiotic prescribing recommendations. These activities were opportunistic and occurred during the usual rostered hours. Interactions were not scheduled and no formal documentation of AD encounters was made. Posters and laminated cards with information about severity assessments and appropriate antibiotic choices for patients with CAP were distributed and actively promoted throughout the ED during the academic detailing period. These personnel and advertising material remained available throughout the following ('computerised decision support') time period, but were not specifically promoted.

At the commencement of the computerised decision support period, the guideline for the management of patients with CAP was deployed on an existing decision support tool. This tool is a web-based transferable system that was designed at the hospital using a .NET framework and implemented in January 2005.

The CAP algorithm used the Pneumonia Severity Index (PSI) to guide site of management decisions (inpatient vs. outpatient care) and the modified British Thoracic Society severity score (CURB) to highlight patients with severe pneumonia who were likely to need review by the intensive care unit (ICU) staff. [17,18] The program was integrated with hospital databases containing patient demographics and pathology results to facilitate rapid calculation of scores required for these prediction rules. Use of these scores was not, however mandated. Users could choose to skip the score to obtain antibiotic advice alone. Antibiotic allergy reminders were included. If a user had previously registered an allergy for a patient this was presented, otherwise a reminder was given to check with the patient. Detailed information was included about unusual pathogens to consider, the most appropriate choice of empiric antibiotics, the duration of therapy, and the timing of change from intravenous to oral antibiotic therapy. Users had access to medical literature via the Internet, along with local interpretation of this literature within the CDSS. Users could browse the CDSS content without logging a patient in, so it could be used as an educational tool as well as providing patient specific advice. There was general agreement between the empiric antibiotic recommendations made in the national guideline, the AD directives and the content of the CDSS.

The CDSS was available hospital wide and its use was entirely voluntary. All hospital clinicians could access it via a shortcut on the desktop of any hospital computer. No specific incentives were provided to encourage its use. It was not triggered by any other computer systems. It resided alongside other electronic hospital guidelines. An introductory demonstration was provided to the ED staff and to all staff at a hospital grand round. Thereafter, infectious diseases registrars or pharmacists provided demonstrations informally.

### Data collection

All patient presentations to the ED were available for inclusion in the study. Patients were prospectively identified from a database in the ED where the treating doctor already routinely recorded the patient's diagnosis. All patients with a diagnosis of pneumonia, chest infection, lower respiratory tract infection, pleuritic chest pain, cough, shortness of breath, and/or aspiration were identified. Patients were included in the study if they had a new respiratory symptom, a new chest x-ray infiltrate consistent with pneumonia, and if the initial assessment made by the treating doctor was that the patient had pneumonia.

Exclusion criteria included: Age <18 years, immunocompromised patients (corticosteroids ≥ 15 mg prednisolone/day for ≥ 2 weeks, HIV positive with CD4 <200 umol/L, transplant recipients on immunosuppressive therapy), suspected or known severe acute respiratory syndrome (SARS), nosocomial pneumonia (discharged from hospital in the previous 2 weeks, after an admission longer than 48 hours), and/or known suppurative lung diseases such as cystic fibrosis or bronchiectasis.

Data were prospectively collected from the medical history by a single trained research nurse, according to a set of specified rules. A single clinician was assigned to make judgements about any difficult issues, and a random sample of these cases was cross checked with a second infectious diseases physician. This group comprised 5% of the total patient cohort (40 patients). Specific clinical and pathological and radiological data available within the first 24 hours were sought to allow calculation of severity scores. [17,18] Clinicians' comments about suspicion of aspiration, and documentation of known antibiotic allergies were recorded. The time to antibiotic therapy was calculated using the time of presentation, documented electronically by the ED triage nurse, and the time of antibiotic administration as documented on the medication chart by the nurse in ED or on the ward.

Information regarding ongoing antibiotic use was collected. Any antibiotics that were clearly being used to treat a separate infection (as described in the patient's medical record) were not included. Where the duration of treatment after discharge was not recorded, it was assumed to be 5 days. Antibiotic costs were calculated using pharmacy purchasing data. No actual changes in the cost of drugs commonly prescribed for pneumonia occurred over the study period. The admission criteria for ICU were based entirely upon the treating clinician's assessment in all time periods. No protocols or guidelines were enforced. Clinicians were not aware that the study was being conducted. The researchers had no clinical role in the ED over the study period. There were no major changes in the number or composition of staff in the ED, or their responsibilities over the study period. This study was approved by the ethics committee of Melbourne Health. Individual consent from the clinicians or the patients involved was not required.

### Outcome measures

The primary outcome assessed was the prescription of empiric antibiotic therapy that adequately covered the likely pathogens (both typical and atypical) and was concordant with recommendations. This included the combination of a recommended beta lactam (amoxicillin, ampicillin, benzylpenicillin, ceftriaxone, cefotaxime or cefuroxime) plus either a macrolide (erythromycin, roxithromycin, clarithromycin or azithromycin) or doxycycline. The use of moxifloxacin alone was also classed as appropriate. Patients who received additional antibiotics were still classed as appropriate, so long as their antibiotic regimen included the recommended drugs (reflecting that the patients at least received appropriate cover). The possibility that antibiotics were required for other concordant problems was appreciated, and without detailed clinical information, it was not possible to determine if this additional antibiotic use was unnecessary.

A number of secondary outcomes were also examined. For patients who required ICU intervention at any time during their admission, the proportion that were admitted directly from the ED to the ICU was evaluated as a marker of early recognition of severe disease. Similarly, the proportion of patients requiring ICU management at any time during their admission who were initially prescribed the recommended empiric broad spectrum antibiotics for severe pneumonia in the ED was compared. Appropriate therapy for this group was defined as ceftriaxone (or benzylpenicillin plus gentamicin), in combination with either intravenous azithromycin or erythromycin. The use of moxifloxacin alone was also deemed appropriate.

The number of patients prescribed an antibiotic to which they had a documented allergy was examined. The overall pattern of antibiotics prescribed, and the average cost of antibiotics per patient, were assessed in each time period. Finally, the time between presentation to the ED and the administration of antibiotics was recorded.

### Statistical methods

Baseline characteristics of subjects were compared between the three periods using a chi-squared test of homogeneity for categorical variables and analysis of variance for continuous variables. An a priori level of statistical significance of 0.05 was assumed.

The baseline period extended over one year to give an indication of the baseline pattern of change in the rate of concordant prescribing over time, in the absence of any intervention. The academic detailing period included enough patients to detect an improvement in mean concordance from 65% to 75% (188 patients, power = 0.8 and p = 0.05). The computerised decision support period included enough patients to detect an expected further improvement in concordance from 75% to 85% (120 patients, power = 0.8, p = 0.05).

Multivariable logistic models were used to compare the mean proportions of concordance across the three periods, while adjusting for disease severity, age, and suspected aspiration. Secondary outcome measures were assessed in the same way. Specifically, among the patients who required ICU admission, the proportion directly admitted from ED to the ICU, and the proportion administered appropriate broad-spectrum empiric antibiotic therapy, were compared. This was specifically recorded as a measure of the degree of recognition of markers of severe illness, which were a key focus of the guideline content. The proportion of patients with a known antibiotic allergy who received that antibiotic was also compared. Time to antibiotic administration was recorded as a measure of whether the CDSS delayed decision making to any extent.

A time series analysis was performed to evaluate changes in concordance of prescribing over time, covering all three time periods. The rate of concordant prescribing was expected to improve over time. Change in concordance over time was assessed with a binary logistic model, incorporating month of treatment as a continuous variable. The 'expected' proportion of concordant treatment at any given time then plausibly corresponds to a regression line fitted through the data. We hypothesized that the rate of concordant prescribing after the intervention (in the third time period) would be greater than that expected given the observed trend before the intervention (the first and second time periods). Statistical analysis was performed using Stata version 9.0. [19]

## Results

The demographic details of the patients in each of the three time periods are presented in Table 1. During the computerised decision support period (CDSS), patients were generally older than those in the other two time periods (a greater proportion were aged >85 years), and less likely to have received antibiotic therapy prior to presentation. The observed death rate during the CDSS period appeared to be higher than for the other two periods, but this was largely explained by differences in the proportion of patients aged over 85 years, and differences in the number of patients who died in the ED for whom supportive therapy was not thought appropriate

**Table 1:** Patient characteristics

**Variable**	**Baseline**		**Academic detailing**		**Computerised decision support**		**Pvalue**^**#**^
	**N = 392**		**N = 215**		**N = 133**		
Age: median (range)	74	(18–96)	73	(18–98)	79	(18–98)	0.05
Sex Female: n (%)	158	40.0%	100	46.0%	60	45.1%	0.29
Nursing home residents: n (%)	55	14.0%	31	14.0%	18	13.5%	0.97
Suspected aspiration: n (%)	39	9.9%	20	9.3%	7	5.3%	0.25
Antibiotics prior to ED: n (%)	100	25.5%	54	25.1%	18	13.5%	0.01
							
**Known beta lactam allergy n (%)**	42	10.7%	23	10.6%	21	15.8%	0.25
Non-immediate	13		6		15		
Uncertain	21		17		2		
Immediate	8		0		4		
							
**PSI class (%)**							
I	11.9%		13.4%		12.8%		
II	14.5%		17.2%		14.3%		
III	17.3%		14.8%		11.3%		
IV	33.4%	}56.1%	28.8%	}54.4%	31.5%	}61.6%	0.39
V	22.7%		25.6%		30.1%		
							
**CURB **severe n (%)	182	46.4%	96	44.6%	55	41.3%	0.65
ICU admission any time n (%)	26	6.6%	12	5.6%	10	7.5%	0.76
Length of stay – days, median (range)	4	(1–76)	4	(1–51)	4	(1–41)	0.93
Death: Total n (%)	37	9.4%	14	6.5%	21	15.7%	0.15*
Death: (excl. died in ED) n (%)	35/390	8.9%	12/213	6.5%	16/128	7.8%	0.23*
							
**Comorbidities**: (%)							
CCF	20.4		15.3		18.0		0.30
COAD	23.4		15.3		26.3		0.02
Neoplasia	13.7		14.4		16.5		0.73
CRF	11.9		13.0		12.7		0.92
Dementia	13.2		14.4		21.0		0.09
Alcohol	9.6		7.9		5.2		0.27
CVA	18.8		17.6		12.8		0.27
Diabetes	22.2		22.7		19.5		0.76
Age >85	19.6		12.5		27.0		<0.01

* Adjusted for age, # p values calculated using chi squared test for categorical variables and analysis of variance for continuous variables

ED: Emergency department, ICU: Intensive care unit, PSI: Pneumonia Severity Index, CURB: modified British Thoracic Society Severity score, CCF: Congestive cardiac failure, COAD: Chronic obstructive airways disease, CRF: Chronic renal failure, CVA: Cerebrovascular disease

Table 2 details the comparisons in prescribing behaviour over the three time periods. The odds ratio for having received the recommended empiric antibiotic therapy to cover both typical and atypical pathogens ('concordant therapy') in the ED for the academic detailing period compared to the baseline period was 2.58 [1.78, 3.73], p < 0.01, and after adjustment for age, severity (PSI class) and suspicion of aspiration, OR = 2.79 [1.88, 4.14], p < 0.01. The odds ratio for concordant therapy in the computerised decision support period compared to the academic detailing period was 2.03 [1.13, 3.66], p = 0.01, and after adjustment for age, severity and aspiration, OR = 1.99 [1.07, 3.69], p = 0.02. The estimated effect over time within each cohort did not appear to be substantially altered by the inclusion of these covariates.

**Table 2:** Outcomes

**Outcome**	**Baseline group**	**Academic detailing group**	**Computerised decision support group**	**P value**^**#**^
	**N = 392**	**N = 215**	**N = 133**	
Patients receiving recommended antibiotic cover for typical and atypical pathogens*	211/341 61.9%	143/208 68.7%	113/126 89.7%	<0.01
Patients requiring ICU who went direct from ED	17/26 65.0%	9/12 75.0%	8/10 80%	0.68
Patients requiring ICU who received appropriate empiric broad spectrum antibiotics#	12/25 48.0%	5/11 45.0%	9/10 90%	<0.01
PSI class V patients who received appropriate empiric broad spectrum antibiotics#	9/70 12.8%	4/43 9.3%	10/38 26.3%	<0.01
PSI class IV&V patients who received appropriate empiric broad spectrum antibiotics #	30/341 8.6%	9/208 4.3%	5/39 12.8%	<0.01
CURB 'severe' patients who received appropriate empiric broad spectrum antibiotics#	14/155 9.0%	7/80 8.7%	20/49 40.8%	<0.01
Patients who received an antibiotic to which they had a known allergy	11/42 26.2%	6/23 26.1%	3/21 14.3%	0.50
Time from ED presentation to administration of antibiotic: median (range)	171 minutes (15–1969)	158 minutes (15–1154)	142 minutes (10–1190)	<0.01
Average cost of antibiotics for pneumonia per patient	$72.07	$94.47	$84.04	NA

* = excluding patients suspected of having aspirated and patients who received no antibiotic treatment at all

# p values calculated using chi squared test for categorical variables and analysis of variance for continuous variables

ED: Emergency department, ICU: Intensive care unit, PSI: Pneumonia Severity Index, CURB: modified British Thoracic Society Severity score

**Figure 1:**
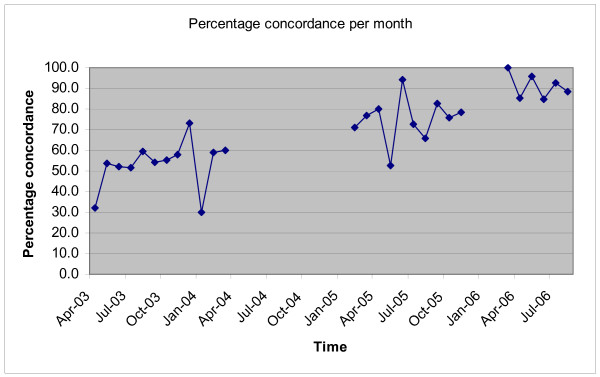
Percentage of empiric antibiotics prescribed that were concordant with recommendations per month.

The effect of change over time was observed in more detail. Figure 1 illustrates the percentage of empiric antibiotic prescriptions that were concordant with recommendations per month over the entire period. Prescribing patterns improved slowly over time. One year after release of the guideline, in the absence of any promotional efforts, (that is, at the end of the baseline period), the concordance rate was around 60%. The change in the proportion of concordant prescribing between the last month of the baseline period and the first month of the academic detailing period was +10.8% over 12 months. The change in the proportion of concordant prescribing between the last month of the academic detailing period and the first month of the computerised decision support period was +21.5% over 5 months. At the end of the study period, the rate of concordant prescribing was high. The first month post the CDSS intervention had a very high concordance rate (100%) and thereafter the rate remained around 90%, although the study was not long enough to demonstrate whether this level was maintained beyond 6 months.

**Figure 2:**
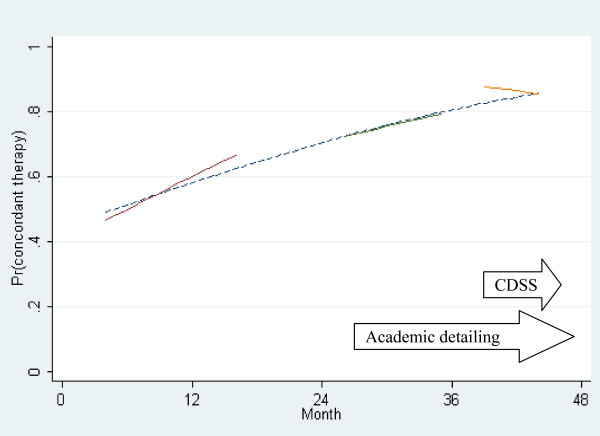
**Proportion of concordant therapy prescribed over time**. The solid lines indicate regression lines that best fit the observed data in each of the three time periods, demonstrating the percentage of empiric antibiotic therapy that was concordant with recommendations per month over time. The broken line is a regression line that best fits the observed data in just the first and second time periods. This line is projected forward over the third time period to demonstrate the 'predicted' concordance if the underlying trend from the first two time periods was to continue. The horizontal arrows demonstrate the timing of the two interventions. The vertical arrow represents the difference between the 'predicted' concordance and the observed concordance after the computerised decision support system (CDSS) intervention.

Further analysis was performed to compare the observed results with that which would be expected based upon an underlying trend in improvement over time [11]. The observed behaviour in the preceding time periods (over 3 years) were used to predict the expected prescribing behaviour in the latter 6 month period of the study. Figure 2 shows the three regression lines that best fit the observed rate of concordance over the three separate time periods, and the concordance predicted from a logistic regression model based upon the first and second time periods extrapolated forward through the third time period (the 'expected' concordance). While it is important to note that such a regression line may be sensitive to outliers, there were in fact few actual outliers in these actual data and the likelihood of effect would be low.

During the first six months of the CDSS period, the proportion of patients who were prescribed concordant therapy was greater than would be expected based on the observed trend. A confidence interval around the trend line was determined, and this described the likelihood of the observed results in the first month of the CDSS period as having a p value of 0.06 based on the existing trend alone.

Secondary outcomes were analysed as a measure of the impact of the changes in prescribing on key areas of interest. Regarding those patients who required ICU support, the likelihood that recommended broad spectrum empiric antibiotics were received in the ED increased over time. The odds ratio for the academic detailing period compared to the baseline period was 1.48 [0.35,6.25], p = 0.59, and the odds ratio for the CDSS period compared to the AD period was 10.80 [0.99, 116.99], p = 0.051. Improved early recognition of patients with severe illness was suggested, with a greater proportion of patients requiring ICU care going directly to ICU from the ED over time (65% in baseline, 75% in the academic detailing period and 80% in the period of computerised decision support). The number of patients was too small to comment upon whether this change was statistically significant.

There appeared to be a lower likelihood of inappropriately prescribing an antibiotic to a patient who had a documented allergy to that drug during the computerised decision support period. Specifically, comparing the AD period with the baseline, the odds of an allergy prescribing error were 0.99 [0.31, 3.16], p = 0.99; whereas when the comparing the CDSS with the AD period, the odds ratio for such a prescribing error was 0.47 [0.10, 2.19], p = 0.33.

Table 3 describes the most frequent antibiotic combinations prescribed to patients in each of the three time periods. The percentage of patients empirically prescribed a cephalosporin was 38.2% for the baseline period, 38.1% for the academic detailing period, and 42.8% for the computerised decision support period. The average cost of antibiotic therapy per patient was calculated for the three patient groups. This calculation was adjusted for changes in pricing over time, though in fact, very little change occurred in the price of the antibiotics most frequently prescribed for CAP during the study. While the average cost per patient increased between the first and second time periods, it fell in the third time period. Finally, the time between a patient being admitted to the emergency department, and an antibiotic being first administered to the patient did not increase, and was actually found to progressively fall over the three time periods, from 171 to 158 and then 142 minutes, p < 0.01.

**Table 3:** The most frequent initial antibiotic combinations prescribed (described as percentage of patients)

Initial antibiotic combination	Percentage of patients
**Baseline period**	
Penicillin IV + roxithromycin	20.6%
Ceftriaxone	10.2%
Ceftriaxone + roxithromycin	9.2%
Penicillin IV	5.8%
Penicillin IV + doxycycline	5.3%
Ceftriaxone + erythromycin IV	4.3%
	
**Academic detailing period**	
Penicillin IV + roxithromycin	27.4%
Ceftriaxone + roxithromycin	16.7%
Amoxycillin (oral)+ roxithromycin	8.3%
Ceftriaxone	6.9%
Roxithromycin	5.1%
Penicillin IV + doxycycline	4.2%
	
**Computerised decision support period**	
Penicillin IV + roxithromycin	28.6%
Ceftriaxone + roxithromycin	17.3%
Ceftriaxone + azithromycin	12.8%
Ceftriaxone	6.7%
Penicillin IV + doxycycline	6.0%

## Discussion

This study demonstrates the pattern of behavioural change in emergency department clinicians over three and a half years, and describes the changes surrounding different interventions to promote a particular prescribing strategy. In particular, it demonstrates that the implementation of a computerised decision support system was associated with greater improvement in prescribing practices than would have been expected based upon the predictions made from actual prescribing observed over the preceding 3 years.

The baseline period provides an example of the rate of change of prescribing behaviour with passive, informal means of information transfer. It shows that change is slow, and that the rate of change falls with time. This is consistent with the suggestion that while some clinicians respond to recommendations early, others may be more difficult to access, or more resistant to change, and change may be harder to achieve in the later time periods.

The improvement in concordance of prescribing was not dramatic with academic detailing, but appeared to be greatest immediately after the CDSS was deployed. It is likely that the interest generated by a novel system, and the attention it received during early education sessions contributed to the high initial concordance. Junior staff in this ED rotated on average every three months, which means that the impact of AD may not be sustained as new staff enter the unit. It is important to note that 100% concordance should not be expected in this context. The CAP guideline represents a basic recommendation, and individual patients vary from the average. In the case of CAP, experienced clinicians would be expected to vary from the guidelines for valid clinical reasons. It is impossible to separate the effect of the computerised decision support system itself, from the effect of the education sessions, which would have increased awareness of the CAP guideline and its recommendations. A longer duration of follow up after deployment of the CDSS would be required to comment upon the sustainability of any change.

The CDSS was associated with changes in many of the secondary outcomes of interest that were not demonstrated with academic detailing. In particular, better recognition of patients with severe pneumonia, suggested by increased use of recommended broad-spectrum empiric antibiotics in those requiring ICU care was noted. This change occurred without a major increase in the overall rate of cephalosporin use or the average antibiotic costs per patient. This may be because the content of the decision support system highlighted this perceived problem, and the advice was consistent for all users. In contrast, with passive transfer and academic detailing advice might be less consistent.

One of the strengths of this paper is that our statistical analysis has taken in to account the expectation that prescribing practices would improve over time, in the absence of intervention. [11] This improvement is presumably due to a 'learning effect' as information is disseminated. It demonstrates that trends in prescribing practices were already present before any specific intervention and these should be acknowledged.

This is one of the first papers to compare the impact of a CDSS with academic detailing alone in the same clinical setting. To date, academic detailing has been one of the more common strategies used to promote guidelines, but it can be a labour intensive exercise. The staff members who provided academic detailing attended a two-day training session, and thereafter dedicated a portion of their clinical time to training purposes. The information provided to different staff members may have varied due to time constraints or the interest of the trainer, and particular areas may not have been discussed. The CDSS, in contrast, provided consistent advice, and could be accessed whenever required by the clinicians. It required an initial investment of clinician's time to develop and test the algorithm, but thereafter did not consume any additional staff resources.

To date, most evaluations of CDSS in hospitals have described large purpose built systems, often in academic centres in the USA with a specific interest in computerisation. [8,20] This paper, in contrast, describes a transferable web based computerised decision support system which can be integrated with many existing clinical databases in other hospitals. This study describes a clinical setting that would be familiar to most tertiary Australian hospitals. Previous reviewers have noted the lack of reports of systems outside of the USA, and this paper therefore provides an important contribution. [21]

The major limitation of this study is that the changes were not compared with a separate control group. This study used the same group of clinicians at different time points as controls. In order to do this, the effect of time needed to be taken into account. The predictions of prescribing patterns that we have described are extrapolations beyond the actual data, and make assumptions about patterns of practice remaining similar over time. In this hospital, it would not have been practical to separate control and intervention groups without cross contamination. In addition, such a study might increase clinician awareness and introduce bias affecting prescribing practices. Although multiple testing issues are a concern where several hypothesis tests are performed, in this study the findings comparing time periods were relatively consistent across different variables and the statistical significance of the effect was generally better than the 0.05 level.

It is also important to recognize that the successful implementation of CDSS depends heavily on the personnel and the setting, hence separate hospitals or wards do not necessarily provide accurate control groups for comparison. The 'culture' within an institution has important effects on guideline implementation strategies. Exploration of the effect of a computerised decision support system on the prescribing practices in other institutions would, therefore, be of interest.

## Conclusion

This study has demonstrated improved antibiotic prescribing practices in a hospital setting associated with two different strategies for implementation of guidelines. The improvement in prescribing practices was initially more significant with computerised decision support system than with academic detailing alone, although this may represent the effect of increased attention being given to a novel system. Further exploration of the role of computerised decision support system in hospitals is warranted to particularly to assess the sustainability of the effect on clinician decision-making at the point of care.

## Competing interests

The authors declare no financial conflict of interest. All authors have been employed by Melbourne Health who now hold the rights to the computerised decision support system evaluated in this study. Melbourne Health had no influence over the findings described in this study. The authors have no other personal financial interests in the CDSS.

## Authors' contributions

KB and KT designed the study, carried out data collection and data analysis. JB and LM provided specific advice regarding statistical evaluation at the study design and analysis stages. AS GB and MK participated in study design and analysis. All authors contributed to the final manuscript.

## Pre-publication history

The pre-publication history for this paper can be accessed here:


